# Posttransplant Lymphoproliferative Disease Presenting as an Extracranial Mass

**DOI:** 10.1155/2017/6401086

**Published:** 2017-10-11

**Authors:** Reuben J. Arasaratnam, Alejandro Restrepo

**Affiliations:** Department of Medicine, Section of Infectious Diseases, Baylor College of Medicine, Houston, TX, USA

## Abstract

Posttransplant lymphoproliferative disease is a serious complication following stem cell and solid organ transplantation. Early recognition of the disease is important in facilitating timely therapy and improving long-term outcomes. We report a renal transplant recipient presenting with an extracranial frontoparietal soft tissue mass that was subsequently diagnosed as a B-cell lymphoma. The patient was treated successfully with immunosuppression reduction, anti-CD20 monoclonal antibody therapy, and cytotoxic chemotherapy. Our case highlights the importance of recognizing soft tissue masses in the head and neck as a potential clinical manifestation of PTLD in solid organ transplant recipients.

## 1. Introduction

Posttransplant lymphoproliferative disease (PTLD) is a serious immunosuppressive-related complication of patients following solid organ or stem cell transplantation with a reported incidence between 1 and 25% [1–4] and mortality as high as 50% [5, 6]. Identifying patients with PTLD remains challenging not least because of the variety of initial clinical manifestations. These range from nonspecific presentations such as fever, weight loss, and night sweats to lymphoma-like masses in native organs and even overt sepsis [7, 8]. Soft tissue manifestations of PTLD at extracranial sites are rare and if not recognized in a timely manner can result in delay of diagnosis and treatment. In this case, we describe a patient presenting with a forehead mass nine months following renal transplantation that was subsequently diagnosed as a B-cell lymphoma (PTLD) and successfully treated.

## 2. Case Presentation

**Figure 1:**
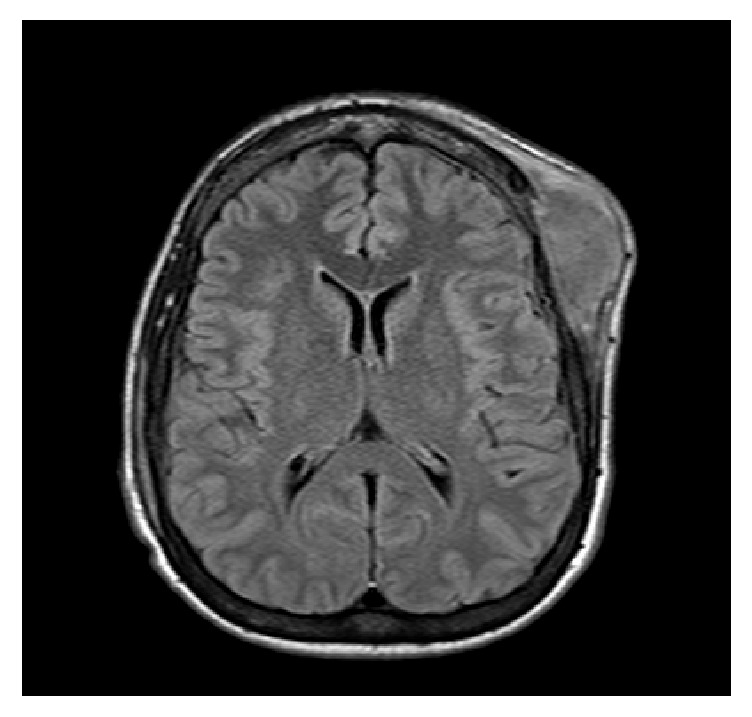
MRI of the brain displaying left frontoparietal dominant extracranial soft tissue lesion.

**Figure 2:**
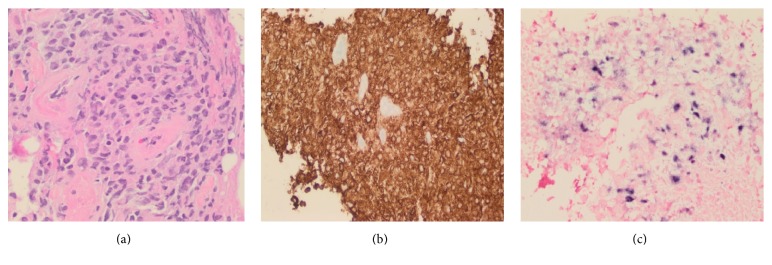
Biopsy of this lesion confirmed an EBV positive B-cell lymphoma. (a) Histology showed large atypical lymphocytes; hematoxylin and eosin (H&E) (×40 magnification). (b) Immunostaining revealed that these lymphocytes were CD20 positive. (c) In situ hybridization with EBV-encoded small RNA (EBER) was additionally positive (×40 magnification).

A 24-year-old man with end-stage renal disease secondary to hypertension underwent a cadaveric renal transplant (donor Epstein-Barr virus (EBV) IgG positive, recipient EBV IgG negative) with basiliximab induction and maintenance immunosuppression consisting of tacrolimus, mycophenolate mofetil, and prednisone. Nine months after the transplant, he presented to the clinic complaining of a forehead mass that had been present for four weeks. He ascribed the development of this mass to mild head trauma sustained previously when he fell out of bed. He denied neurological or constitutional symptoms. His physical examination was notable for a golf-ball-sized mass in the left frontoparietal region that was firm in consistency, nonmobile, with no overlying skin abnormality. There were no neurological abnormalities, hepatosplenomegaly, or peripheral lymphadenopathy. Further imaging of the mass was ordered with an MRI of the brain which showed focal cranial bone marrow infiltration and a left frontoparietal 6 × 2 × 9 cm dominant extracranial soft tissue lesion (Figure 1) with thickened enhanced dura below this site. A complete blood count, comprehensive metabolic panel, blood cultures, and urinalysis were unremarkable. A core biopsy of the mass revealed large atypical lymphocytes (Figure 2(a)) that stained positive for the B-cell marker CD20 (Figure 2(b)), with a high Ki-67 proliferative index and positive EBER staining (detecting for in situ EBV replication) (Figure 2(c)).

Cerebrospinal fluid studies were negative for malignant cells. Further staging imaging with a contrast-enhanced CT of the chest, abdomen, and pelvis showed necrotic retroperitoneal lymphadenopathy. A serum EBV viral load performed was elevated at 75,000 copies/ml. A final diagnosis of Epstein-Barr virus (EBV) positive B-cell lymphoma was made. Mycophenolate mofetil was stopped. The patient was considered to be at high risk for central nervous system disease and received a single dose of prophylactic intrathecal cytarabine. He underwent his first cycle of R-CHOP (rituximab, cyclophosphamide, doxorubicin, vincristine, and prednisone) as an inpatient and was monitored closely for treatment toxicities. Aside from a chemotherapy-related neutrophil nadir of 800 neutrophils/microliter which recovered quickly with growth factor support (granulocyte-colony stimulating factor), he suffered no adverse treatment-related events. By the end of his first cycle of R-CHOP, the forehead mass had decreased mildly in size and his serum EBV viral load had declined to 20,400 copies/ml. He went on to complete a further 6 cycles of R-CHOP (total of 7 cycles) and achieved remission 6 months later. Transplant immunosuppression was maintained with tacrolimus and prednisone both during and following his chemotherapy, and renal allograft function remained normal with no acute rejection events.

## 3. Discussion

The necessary use of long-term immunosuppression following solid organ transplantation (SOT) is associated with a number of infectious and noninfectious complications, including PTLD. PTLD represents a spectrum of clinical disorders due to lymphoid hyperproliferation (most often B-cell in origin), ranging from a benign hyperplasia to an aggressive malignant lymphoma [9]. In approximately 50% of cases [10, 11], Epstein-Barr virus plays an oncogenic role by inducing transformation and proliferation of B-lymphocytes, which continues unchecked when the EBV-specific cytotoxic T-cell response is impaired due to iatrogenic immunosuppression [12]. Consequently, solid organ transplant patients at the highest risk of PTLD include EBV-seronegative recipients of an allograft from an EBV-seropositive donor and those receiving high-intensity immunosuppression including lymphocyte depleting therapies [13]. The clinical presentation of PTLD most frequently involves extranodal sites such as the gastrointestinal tract, lungs, central nervous system, and the transplanted allograft [14]. Skin and soft tissue presentations of PTLD have also been described. These include nodules, ulcerative lesions, and plaques that are characteristically localized to the extremities, trunk, and face [15]. To our knowledge, only two prior presentations of PTLD in adult SOT recipients, presenting as forehead soft tissue masses, have been described in the literature [16, 17]. Importantly, these extracranial lesions could potentially be mistaken for a benign or trauma-related mass resulting in diagnostic delay. Regardless of the clinical presentation, a definitive diagnosis of PTLD requires biopsy and comprehensive analysis of the tumor tissue including histopathology for functional architecture, immunophenotyping to characterize the predominant lymphocyte subset, and detection of EBV in the tissue using in situ hybridization with an EBV-encoded RNA probe (EBER-ISH) [18].

The mainstay of treatment for PTLD is the reduction of immunosuppression which has led to variable response rates of between 6 and 48% [19–21]. Not all patients can tolerate or respond to immunosuppression reduction and this approach increases the risk of allograft rejection which has been reported to be as high as 32–39% [19, 20]. If reduction of immunosuppression is unsuccessful, the most frequently employed therapeutic modalities are the use of rituximab (monoclonal anti-CD20 antibody) and combination chemotherapy with cyclophosphamide, doxorubicin, vincristine, and prednisone (CHOP) [22, 23]. Work by Trappe and colleagues, looking at the treatment of CD20-positive PTLD in solid organ transplant recipients, showed that high remission rates can be achieved by using sequential therapy with rituximab followed by chemotherapy with CHOP [24]. In this multicenter prospective trial, solid organ transplant recipients with CD20+ PTLD received 4 cycles of rituximab followed by 4 cycles of CHOP and achieved remission rates (complete or partial) of 90%, with treatment-related mortality of 11%. A further modification of this approach, using the initial response to rituximab therapy to guide further consolidation therapy with rituximab alone or rituximab with CHOP chemotherapy, was recently published by these same authors, showing that select patients can achieve sustained responses with single agent rituximab therapy, avoiding chemotherapy altogether and its associated toxicities [25].

The use of antiviral drug therapy for preventing or treating PTLD remains controversial. Lytic EBV replication can be inhibited in vitro by the guanine nucleoside analogs acyclovir and ganciclovir [26, 27]. However, these antiviral drugs require monophosphorylation by EBV thymidine kinase prior to being incorporated into viral DNA. The limited expression of EBV-encoded thymidine kinase in latently transformed B-cells renders these drugs of limited therapeutic value in vivo when PTLD is established [28]. Furthermore, a large recent systematic review showed no prophylactic benefit of these antiviral agents for the prevention of PTLD in high risk (EBV-naïve) pediatric and adult solid organ transplant recipients [29]. Cidofovir and foscarnet are broad-spectrum antiviral medications including activity against EBV. The mechanism of action of these drugs, directly inhibiting the viral DNA polymerase (without the need for prior phosphorylation), provides a strong rationale for their use in PTLD. However, to date, success of these antiviral drugs used either alone or in combination with intravenous immunoglobulin to treat PTLD in SOT recipients has been limited to case reports and small case series [30–32].

An alternative and promising therapy is the use of adoptive transfer of EBV-specific cytotoxic T-lymphocytes, which has been successful in a number of studies for the treatment and prevention of PTLD in allogeneic stem cell transplant recipients and to a lesser degree in solid organ transplant recipients [33–35]. Haque and colleagues conducted a phase II multicenter trial in which allogeneic EBV-specific cytotoxic T-lymphocytes (matched at 2 to 5 HLA alleles) were administered to 31 solid organ transplant and 2 stem cell transplant recipients with PTLD who had failed initial conventional therapy [36]. Following administration, there were no immediate infusion-related events or episodes of allograft rejection. Response rates of 64% and 52% were seen at 5 weeks and 6 months, respectively. Although these immunotherapies have characteristically been limited to the research setting, recent developments simplifying the manufacturing process [37] and establishing banks of virus-specific T-cells enabling an “off-the-shelf” use [38, 39] may serve to increase the availability of this therapy in the future to treat refractory viral infections (including PTLD) in transplant recipients.

As a strong association between EBV and PTLD exists, detection of the EBV genome (in the form of quantitative polymerase chain reaction of EBV DNA from peripheral blood) has been proposed as a potential screening strategy to guide therapeutic interventions to prevent the development of PTLD [40]. Lee et al. implemented a protocol in their center to evaluate the benefit of EBV viral load driven reduction of immunosuppression on the incidence of PTLD in 73 pediatric liver transplant recipients [41]. They prospectively monitored EBV viral load in the posttransplant setting and used a threshold of 4000 copies/microgram DNA on two consecutive measurements to trigger a decrease in tacrolimus dosing (to a trough goal 4–6 ng/ml) and cessation of steroids. Using this protocol, they found a dramatic reduction in the incidence of PTLD from 16% (preintervention) to 2% (postintervention). Importantly, out of the 11 patients who underwent immunosuppression reduction, only one patient developed acute allograft rejection which was successfully managed with steroid pulsing and cessation of tacrolimus tapering and no requirement for retransplant. More recently, Choquet et al. designed a protocol whereby EBV viral load thresholds of 10^5^ and 10^6^ copies/ml were used to guide not only reduction of immunosuppression (stopping mycophenolate mofetil and reducing cyclosporin dose) but also administration of single-dose rituximab (375 mg/m^2^) in 299 adult heart transplant recipients [42]. Following implementation of this protocol, they also found a significant decrease in the incidence of PTLD compared to a historical control group and no significant increase in the risk of allograft rejection. Other successful interventions based on EBV viral load monitoring that have been described include the combined use of antivirals and immunosuppression reduction [43] and the infusion of autologous EBV-specific cytotoxic T-lymphocytes [44]. Together, these studies allude to the potential benefit of using EBV viral load monitoring in the posttransplant setting to guide interventions to prevent the development of PTLD. However, before such practices can be widely adopted, further research is needed to identify the optimal approach to viral load monitoring (including assay, screening interval, and action threshold), the relative benefits and risks of different interventions, and overall cost-effectiveness of such an approach [18, 45].

In summary, PTLD is a rare but serious complication of solid organ transplantation. Awareness of the potential clinical manifestations of this disease is important in making an early diagnosis. Our case highlights the importance of considering PTLD in the differential diagnosis of a transplant recipient presenting with a soft tissue extracranial mass.
